# Vortex bifocusing of extreme ultraviolet using modified Fermat-spiral photon-sieve splitter

**DOI:** 10.1515/nanoph-2024-0389

**Published:** 2024-09-24

**Authors:** Yuanyuan Liu, Huaiyu Cui, Yujie Shen, Yongpeng Zhao, Shumin Yang, Gangwei Wang, Xin Tong, Junyong Zhang, Qiwen Zhan

**Affiliations:** School of Optical-Electrical and Computer Engineering, 47863 University of Shanghai for Science and Technology, Shanghai 200093, China; Zhangjiang Laboratory, 100, Haike Road, Shanghai, 201204, China; National Key Laboratory of Laser Spatial Information, Harbin Institute of Technology, Harbin 150080, China; Shanghai Synchrotron Radiation Facility, Shanghai Advanced Research Institute, Chinese Academy of Sciences, Shanghai 201204, China; Key Laboratory of High Power Laser and Physics, Shanghai Institute of Optics and Fine Mechanics, Chinese Academy of Sciences, Shanghai 201800, China

**Keywords:** extreme ultraviolet focusing, vortex beams, photon-sieve splitter

## Abstract

Structured beams carrying orbital angular momentum (OAM) provide powerful capabilities for applications in optical tweezers, super-resolution imaging, quantum optics, and ad-vanced microparticle manipulation. However, it is challenging for generate and control the OAM beams at the extreme ultraviolet (EUV) region due to the lack of suitable wave front shaping optics arise from being limited to the strong absorption of most materials. Here, we use a modified Fermat-spiral photon-sieve splitter to simultaneously generate two focused doughnut beams with opposite helical phase. Our technique enables us to produce splitting focused vortex beams with different rotation directions at EUV wavelengths. Additionally, we provide experimental evidence showcasing the capabilities of our method and further detect the helical phase by self-reference interferometry. This work not only opens a route for OAM-driven applications in EUV radiation, but also paves the way to studies of holographic technique by EUV splitter.

## 1 Introduction

Advances in the generation of short wavelengths, especially in the extreme ultraviolet (EUV) realm [1], [2], provide powerful new capabilities in material science [3], attosecond spectroscopy techniques [4], lithography [5] and high-resolution imaging [3]. However, strong absorption of EUV radiation in most optical materials hinders the development of refractive lenses in this spectral region, which makes practical implementation challenging. Many forms of manipulating waves that had substantial impact on applications in the visible regime have remained out of reach in the EUV radiation. Optical vortices, which carry orbital angular momentum (OAM), provide powerful capabilities for extensive application in particle manipulation [6], super-resolution imaging [7] and quantum optical communications [8]. Such vortex beams can be flexibly produced, measured and manipulated with infrared and visible light [9], [10], [11], enabling it as a tool for controlling light–matter interactions or elucidating electron dynamics in physics. Therefore, generating and manipulating OAM in EUV regime and other short wavelengths is an active field of research, which opens an important channel to link mature visible light shaping devices to EUV radiation. Recent advance of producing EUV vortex beams via high-harmonic generation [12], [13], synchrotron undulator radiation [14] and laser-seeded free electron lasers [15], have opened up the possibility of monitoring and manipulating the OAM of light–matter interaction on the atomic scale. However, most optical materials strongly absorb light in this wavelength regime, resulting in a scarcity of appropriate optical devices for wavefront shaping, such as spatial light modulators or any transmissive components. This limitation hinders the ability to impart OAM applications in EUV radiation.

To date, refractive elements have so far been missing in the EUV wavelengths, and the optics for focusing have been limited to reflective components or diffractive Fresnel zone plates (FZP) [16], [17]. Therefore, the effective focusing of EUV waves is especially challenging. For example, Cappsso. et al. designed a metalens to focus EUV light. However, this kind of dielectric metasurfaces with ultra-high-precision machining is only accessible using state-of-the-art electron-beam etching [18]. Recently, we demonstrated that the self-evolutionary photon sieves can be effectively used to focus and shape EUV light, which opens a route for controlling and shaping of complex focused beams for short wavelengths [19]. Soon afterwards, the anamorphic Fermat-spiral photon sieves (PS) was used to produce structured-focusing EUV OAM beams with controllable topological charges, which opens a route for enlarging OAM applications in EUV regime [20].

Here, we propose a different strategy, implement this concept by designing the modified Fermat-spiral photon-sieve splitter to generate splitting focused vortex beams with nanoscale focal spot and millimeter-scale focal lengths in EUV radiation. The proposed photon-sieve splitter enables the manipulation of splitting focused vortices, allowing for the control of topological charges and rotational directions by varying the structure of the PS. Photon-sieve splitter in this case refers to a wave shaping device that utilizes a PS to divide EUV beams into two spatially separate beams. In this article, for the first time we demonstrated that two vortex beams with equal OAM values and opposite helicity can be generated and detected simultaneously in the EUV region using a 46.9 nm light source. The proposed method not only provides the possibility of creating two vortex beams with opposite equal helicity in EUV radiation, but also can serve as optical tweezers by optical traps interference to manipulate cellular structures at the atomic scale, leveraging their short wavelength advantages. In addition, we also demonstrate the use of holography principle to detecting the OAM of light with a specific topological charge, providing the foundation for general-purpose manipulate optics for EUV radiation, such as holographic interfaces and splitting device. In this way, we extended the functionality of a PS for EUV radiation, enabling the manipulating of the various incident states of light, which can provide a wealth of new application opportunities in various areas.

## 2 Design and simulation

**Figure 1:**
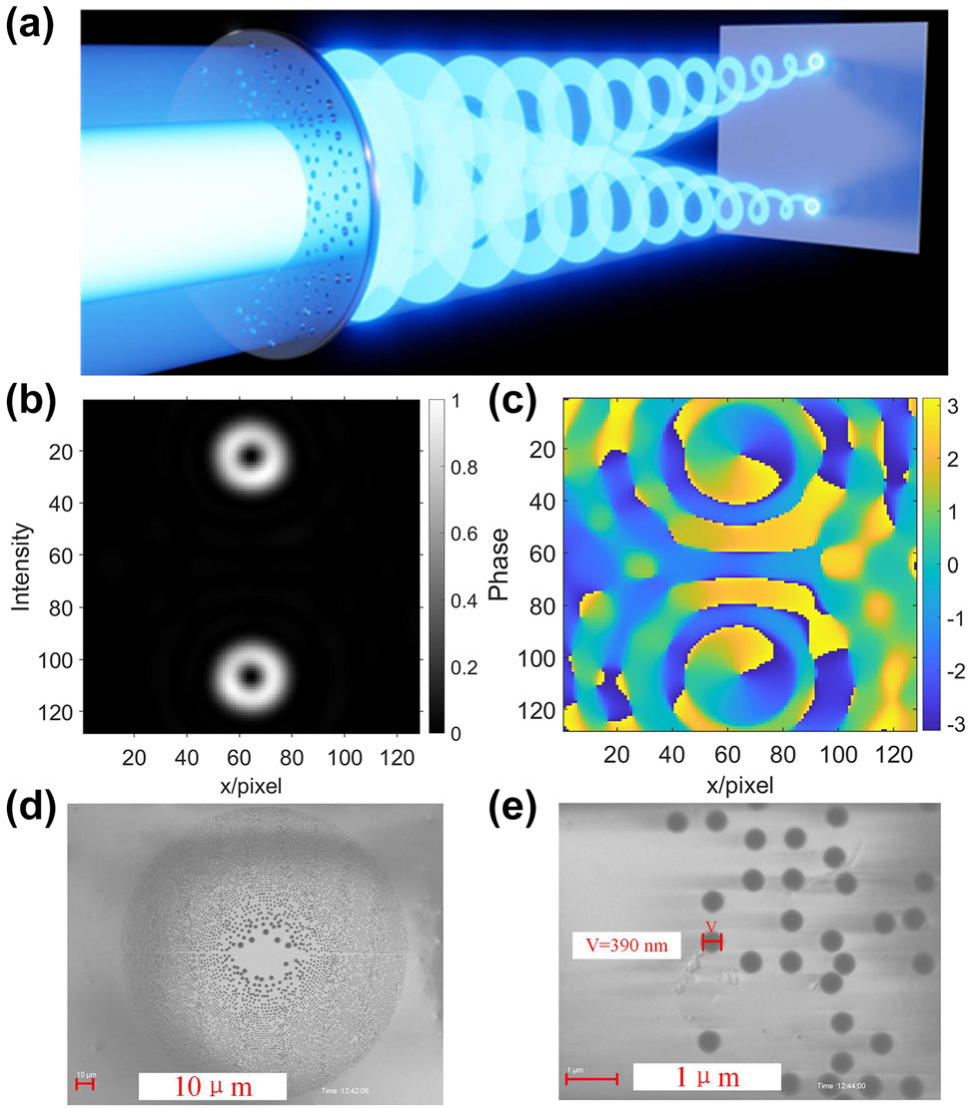
EUV vortex bi-focusing created by a modified Fermat-spiral photon-sieve splitter. (a) Schematic of focused double vortex beams; (b) and (c) simulations of the intensity and phase distribution of two focused vortices with topological charge *l*_1_ = 1 and *l*_2_ = −1; (d) scanning electron microscopy images of vortex splitter fabricated by electron-beam lithography; (e) zoomed-in SEM picture of (d).

As a first demonstration of this novel concept, two bicircular vortex beam field with *l*_1_ = 1 and *l*_2_ = −1 were proposed, to achieve the generation of splitting focused vortices in EUV. The concept of our PS splitter is illustrated in Figure 1(a). In this configuration, a focused beam consisting of doublets of EUV vortex beams is generated, where the beams in each doublet possess the same topological charge, but opposite helicities. Theoretically, the proposed method can provide the OAM selectivity, allowing us to realize different OAM values (including topological charges and twist orientation) for beam shaping and focusing in the EUV regime. Motivated by our recent research work [19], our intent is to provide a way to further broaden the dimensionality of manipulating OAM beams using the PS. In this case, two different focused EUV vortex beams are simultaneously generated, which have different topological charges and rotation directions. Taking into account an incident plane wave, the total optical diffracted distribution of a PS at the focal plane is a summation of those individual diffracted fields from nanostructure [21]. In the design process, Fermat spiral PS can be employed as the foundational distribution for the purpose of structural optimization, and then the genetic algorithm (GA) is developed to optimize the huge number of pinholes in a similar way as in reference [22] (see methods [22]), and then obtain a modified Fermat-spiral photon-sieve splitter working at the wavelength of 46.9 nm.

The corresponding simulations are reported in Figure 1(b) and (c). As expected, two focused vortices occur ring-shaped intensity profiles along light propagating, which is signature of their structure feature of carrying OAM. As for the phase, two focused vortices have same topological charges and different helixes. A top-view scanning electron microscope (SEM) image of our vortex splitter is shown in the Figure 1(d). The zoomed-in SEM picture of as shown in Figure 1(e), in which the smallest pinhole size is around 390 nm. Here, the manufacturing nanofabrication process of our designed PS splitter was realized by three main steps. In the first step, a self-supporting silicon nitride (Si3N4) membrane with a thickness of 100 nm was produced from a silicon nitride wafer through the process of Potassium Hydroxide (KOH) etching, and then a 200 nm-thick layer of ZEP520A was spin-coated onto the top of the Si3N4 membrane. Secondly, the designed PS splitter patterns were delineated on ZEP520A resist utilizing a conventional electron beam lithography (EBL) system. After the electron beam exposure, the sample was developed in a mixture solution of methyl isobutyl ketone (MIBK)/isopropyl alcohol (IPA), and rinsed in IPA. Finally, using the defined ZEP520A as the etching mask, the freestanding sieves were performed by fluorine-based reactive ion etching (RIE) to etch the Si3N4 layer in the transparent pinholes.

## 3 Experiment and results

**Figure 2:**
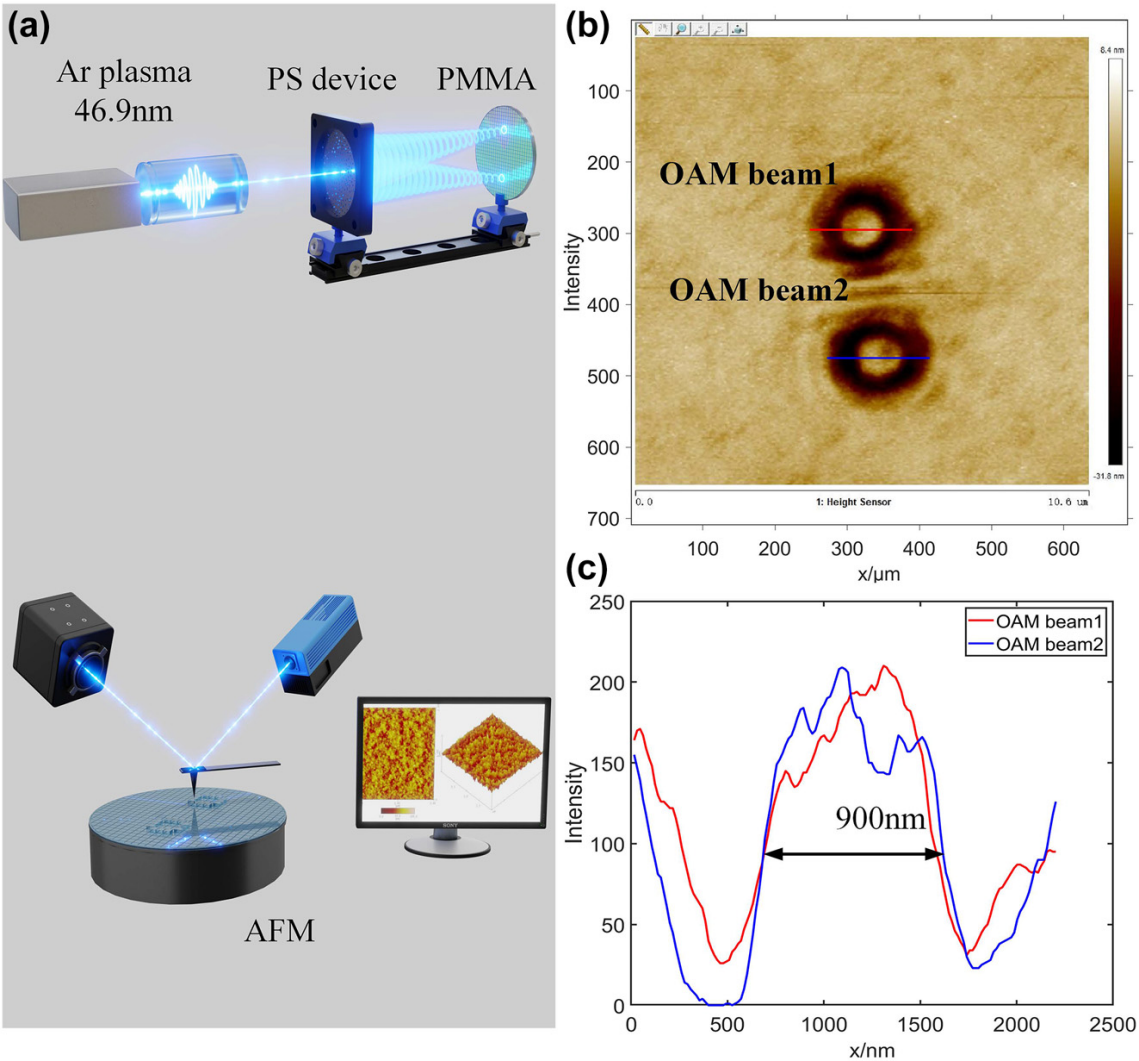
Experimental demonstration of EUV focused splitting vortex. (a) Schematic of the instrument set up including the generating EUV laser optics, focusing and splitting PS and detection medium. Two focused vortex beams were recorded on the PMMA surface, and then their intensity distribution was detected by the AFM; (b) the distribution of raw SEM image of focused double vortex; (c) the intensity curves of the measured double vortex are represented by the red solid line (OAM beam1) and the blue solid line (OAM beam2).

The EUV focusing experiment is schematically shown in Figure 2(a). The EUV radiation is a capillary discharge laser with an output energy of 500 μJ operating at the single-shot mode [23], [24], [25], which a single harmonic order at a wavelength of 46.9 nm is selected. The laser output is collimated and then fully illuminated onto the designed PS vortex splitter, generating a first-order diffracted beam carrying focused OAM of *l* = +1 and *l* = −1. These two vortex beams overlap synchronously in time but completely separate from each other in space. The generated double focused vortex beams undergoes optical transmission in the detection vacuum chamber, and then subjected to detection and analysis. Two vortex beams will be focused on the focal plane of vortex splitter. To record the focal spots with higher accuracy, a polymethyl methacrylate (PMMA, 950,000 molecular weight) with a 200-nm-thick coating on a silicon wafer was used as the recording medium. The PMMA-coated wafer was aligned perpendicular to the beam and placed at a distance of 1.4 mm from the vortex splitter. In the experiment, the PMMA was fixed on a two-dimensional stage and executes *z*-scan along the beam propagation direction, as shown in Figure 2(a). This allowed the intensity distribution of each of two diffracted vortex beams to be collected. Subsequently, the processed PMMA targets are analyzed using atomic force microscopy (AFM), with the profiles obtained from the intensity measurements recorded by the CCD detector. A scanning raw SEM image of the focused double vortex is shown in Figure 2(b). Their spatial intensity distributions are similar, and the central dark core widths are equal, about 900 nm, which is an indication that the absolute values of the topological charges of the double vortices are equal, as shown in Figure 2(c).

**Figure 3:**
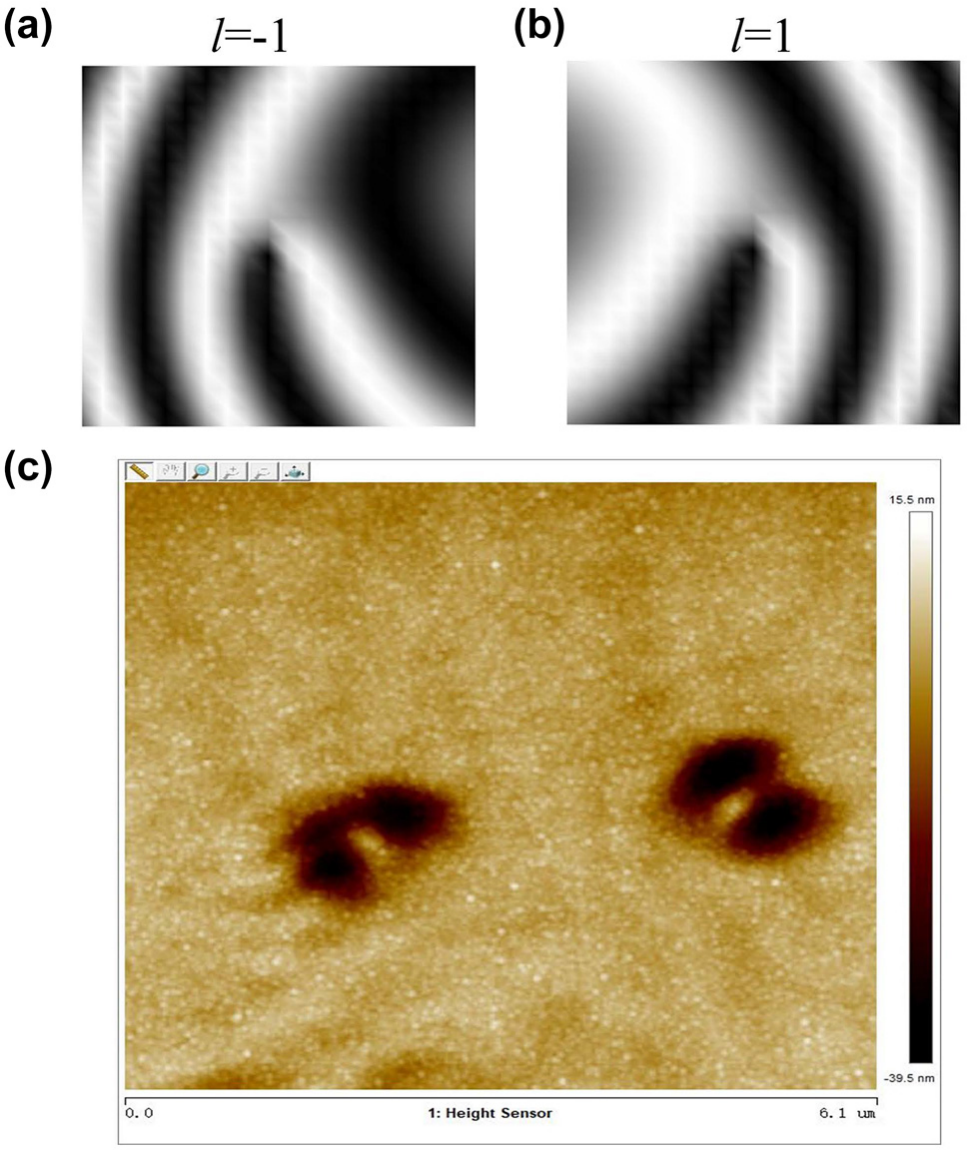
The interference detection of splitting vortex beams. (a), (b) The simulation interference patterns between the plane wave reference beams and focused OAM beams of charges *l*_1_ = −1 and *l*_2_ = 1, respectively; (c) the interference intensity distribution in the experiment.

In order to determine the sign of topological charges of the two vortices, two reference beams are respectively introduced to interfere with them. When two focused vortex beams interfere with a tilted plane wave, an interference pattern can be observed. In this case, the center rotation of the interference pattern is dependent on the sign of the direction of the helices. Thus, the focused EUV double vortex beams can be effectively detected by the distribution of the interference patterns and the direction of fringes. Here, we have simulated the interference patterns between two focused vortex beam with topological charges of *l* = −1 and *l* = 1, and a titled plane wave beam, as depicted in the Figure 3(a) and (b), respectively. Obviously, the shape distribution of interference patterns can reveal the characteristic of the vortex filed, including its topological charge value and rotation direction. Therefore, the value and direction of OAM beams can be easily identified merely with naked eyes under in this optical configuration. Figure 3(c) shows the interference patterns between the reference EUV beams and focused double vortex EUV beams in our experiment. For the two OAM beams, the interference patterns have same singular fringe, which also proves that they have equal absolute topological charge values. In contrast, the interferogram between the reference beam and two focused exhibits different opening directions of the stripes, which indicates the opposite handedness of the helical phase. By comparing the simulation and experimental results, we can find that there is a subtle angular deviation in the rotation of the two interference fringe patterns observed in the experiment. This deviation arises from a minor misalignment between the measurement plane and the focal plane of two focused vortex beams. Nevertheless, it is important to emphasize that the minor deviations encountered during the experimental procedure do not affect the ability to detect the topology and direction of vortex beams utilizing this interference technique, which can also prove the effectiveness and feasibility of our proposed method.

## 4 Conclusions

In this study, we propose and demonstrate a novel technique for the generation of focused EUV splitting vortex beams by utilizing a modified Fermat-spiral PS splitter. As evidence of the proposed method, a special structure light field consisting of double vortex beams was successfully generated, where each vortex has the topological charge value of 1, but opposite helicities. In addition, two vortex beams with equal but opposite topological charges can form a petal-like optical intensity distribution through the linearly superposing, which can be employed for stably trapping microparticles as optical tweezers. The intensity distribution maps of these double-helical wavefronts, characterized by an azimuthal phase, were confirmed in the experiment consistent with the theoretical predictions. Additionally, an interference experiment was conducted to analyze the wavefront properties and helical orientations, revealing opposite helix directions for the two split vortex beams. Our proposed method, by employing a modified spiral PS, offers a groundbreaking approach for the generation of focused splitting vortex beams, which has significant potential applications in nanoscale-manipulation of EUV optical tweezers. More importantly, the proposed vortex splitter in EUV regime in this work is universal and can be extended to broad applications including EUV lithography, holographic interfaces and super-resolution imaging. As a result, our work paves the way for the research on shaping or beam splitters within EUV systems that are currently difficult to investigate.
